# Editorial Comment to Utility of whole‐body diffusion‐weighted magnetic resonance imaging in the management of treatment‐related neuroendocrine prostate cancer

**DOI:** 10.1002/iju5.12248

**Published:** 2020-12-15

**Authors:** Soichiro Yoshida, Yasuhisa Fujii

**Affiliations:** ^1^ Department of Urology Tokyo Medical and Dental University Tokyo Japan

Neuroendocrine prostate cancer (NEPC) is a rare and aggressive malignancy that requires careful monitoring, as it often leads to visceral and osteolytic metastases as well as low prostate‐specific antigen (PSA) production. Studies concerning castration‐resistant prostate cancer (CRPC) patients have reported radiographic progression in 24.5% of cases without clinical or PSA progression when androgen receptor‐axis‐targeted therapies were used, and the utility of follow‐up imaging extends beyond NEPC alone.^1^ In this report, Kurashina *et al*. reported a case with treatment‐related NEPC (t‐NEPC) in which a whole‐body magnetic resonance imaging (WB‐MRI) was useful in monitoring disease progression and treatment response in t‐NEPC with radiographic progression in the absence of PSA progression.^2^ The patient’s osteoblastic metastases responded well to primary anti‐androgen therapy, showing an osteosclerotic response. As distinguishing between osteosclerotic progression and responses in bone metastases is challenging, monitoring bone sclerotic lesions are similarly difficult with bone scintigraphy or computed tomography (CT). WB‐MRI is a next‐generation imaging modality for prostate cancer, as is positron emission tomography‐CT/MRI with prostate‐specific membrane antigen.^3^ Diffusion‐weighted imaging (DWI) is ideal for diagnosing active bone metastases, as both osteoblastic and osteolytic metastases can be visualized as clear, high‐signal images. Moreover, WB‐MRI is a one‐step staging tool, assessing primary prostate cancer as well as lymph node, visceral, and bone metastases simultaneously.^4^ Monitoring as part of the evaluation of treatment response may enable early changes in systemic treatment methods that previously produced no response, as in the present case. DWI signals reflect treatment‐induced changes in the microstructure of cells and can serve as an imaging biomarker when assessing therapeutic response.^5^ Therefore, WB‐MRI holds promise for screening and monitoring systematic metastases in t‐NEPC patients. WB‐MRI may be useful for customizing therapeutic approaches to CRPC as well as t‐NEPC.

## Conflict of interest

The authors declare no conflict of interest.
